# Multiple gastric cancer tissue proteomic identification predicts CLU as a biomarker for anti-PD-1 immunotherapy

**DOI:** 10.1016/j.jpha.2026.101615

**Published:** 2026-04-01

**Authors:** Yu Zhang, Hao-Yi Zhu, Cong-Cong Ma, Ning Wang, Yan Li, Guo-Liang Lu, Xing-Jie Dai, Bo Wang, MAA Mamun, Ying Li, Xiao-Ying Zhao, Jing-Ru Pang, Ning-Jie Guo, Feng-Yu Qi, Jian-Gang Sun, Hong-Min Liu, Feng-Wu Liu, Piet Herdewijn, Long-Fei Zhao, Yi-Chao Zheng

**Affiliations:** a State Key Laboratory of Metabolic Dysregulation & Prevention and Treatment of Esophageal Cancer, Key Laboratory of Advanced Drug Preparation Technologies, Ministry of Education, China, Key Laboratory of Henan Province for Small Molecule Drug Discovery and Application, School of Pharmaceutical Sciences, Zhengzhou University, Zhengzhou, 450001, China; b XNA Platform, School of Pharmaceutical Sciences, Zhengzhou University, Zhengzhou, 450001, China; c School of Chinese Medicine, The University of Hong Kong, Hong Kong, 999077, China; d Department of Biomedicine and Medical Diagnostics, School of Science, Auckland University of Technology, Auckland, 1010, New Zealand; e Maurice Wilkins Centre, The University of Auckland, Auckland, 1142, New Zealand; f Auckland Cancer Society Research Centre, Faculty of Medical and Health Sciences, The University of Auckland, Auckland, 1142, New Zealand; g Department of Gastrointestinal Surgery, The First Affiliated Hospital of Zhengzhou University, Zhengzhou, 450000, China; h Rega Institute for Medical Research, Medicinal Chemistry, Leuven, 3000, Belgium; i School of Biomedical Engineering, Shanghai Jiao Tong University, Shanghai, 200240, China

## Abstract

• Clusterin (CLU) is a new negative predictive biomarker for anti-PD-1 immunotherapy of gastric cancer (GC).
• CLU is a pivotal regulator of both tumor-intrinsic proliferation and immune evasion in GC.
• CLU abrogation in GC enhances the immune response, boosting T-cell killing ability activity and cytokine secretion.

## Graphical abstract

**Figure:**
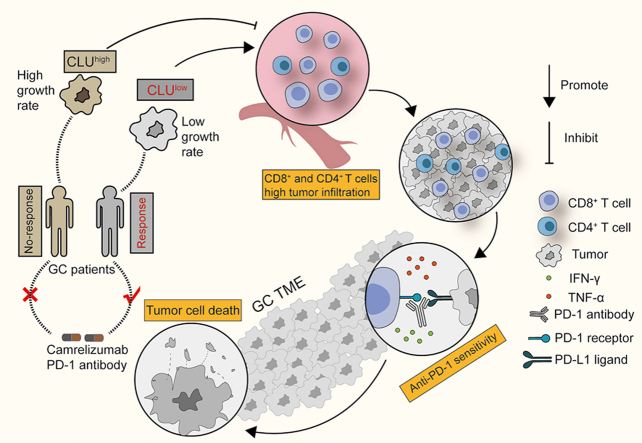

Gastric cancer (GC) remains a leading cause of cancer-related mortality worldwide. Despite improvements in chemotherapy, radiotherapy, and surgery, the prognosis of advanced GC patients remains poor [1]. Recently, immune checkpoint blockade, particularly targeting the programmed death-1 (PD-1)/programmed death ligand-1 (PD-L1) pathway, has provided new therapeutic opportunities [2]. However, the response rate to PD-1 blockade in GC is limited, underscoring the urgent need for reliable biomarkers that can predict treatment efficacy and guide patient selection. Current biomarkers, including PD-L1 expression, microsatellite instability (MSI), and tumor mutational burden (TMB), provide only partial predictive power [3]. Thus, identifying novel biomarkers that reflect tumor–immune interactions is a major clinical priority.

In this study, we performed comparative proteomic profiling of pre-treatment tumor tissues from 28 advanced GC patients treated with camrelizumab (anti-PD-1 antibody). And the detailed workflow is illustrated in Fig. 1A. Based on response evaluation criteria in solid tumor, 17 patients were classified as responders (complete response and partial response) and 11 as non-responders (progressive disease and stable disease). Patient demographics and baseline clinical characteristics are provided in Table S1. Proteomic analysis identified 118 significantly altered proteins between these two groups, with 51 upregulated and 67 downregulated in non-responders (Fig. 1B, fold change > 1.5, *P* < 0.05). Integration with RNA sequencing datasets from two independent anti-PD-1-treated melanoma cohorts revealed that clusterin (CLU), a stress-associated glycoprotein implicated in tumor progression and therapy resistance [4,5], was the only gene consistently upregulated in non-responders across all three datasets, whereas no genes showed a similarly consistent pattern of downregulation (Figs. 1C and S1). Receiver operating characteristic (ROC) analysis further validated CLU as a predictive biomarker for anti-PD-1 therapy efficacy in GC, with an area under the curve of 0.834 (Fig. 1D). To further explore the link between CLU expression and therapeutic efficacy, immunohistochemical (IHC) staining analysis was performed on tumor tissues from the 28 GC patients, before treatment with camrelizumab, focusing on CLU and PD-L1 (a known positive biomarker for anti-PD-1 therapies [3]). IHC staining of GC tissues validated the proteomic results. CLU expression levels were significantly higher in non-responders, whereas PD-L1 failed to discriminate between groups (Figs. S2A and B). Moreover, tumor microenvironment (TME) analysis using the TIMER2.0 database revealed that high CLU expression was positively correlated with immunosuppressive cells such as cancer-associated fibroblasts, M2 macrophages, and regulatory T cells, and negatively correlated with immune-activating cells including natural killer (NK) cells and M1 macrophages (Fig. S2C). These findings suggest that CLU contributes to an immunosuppressive TME and resistance to PD-1 blockade.

**Fig. 1:**
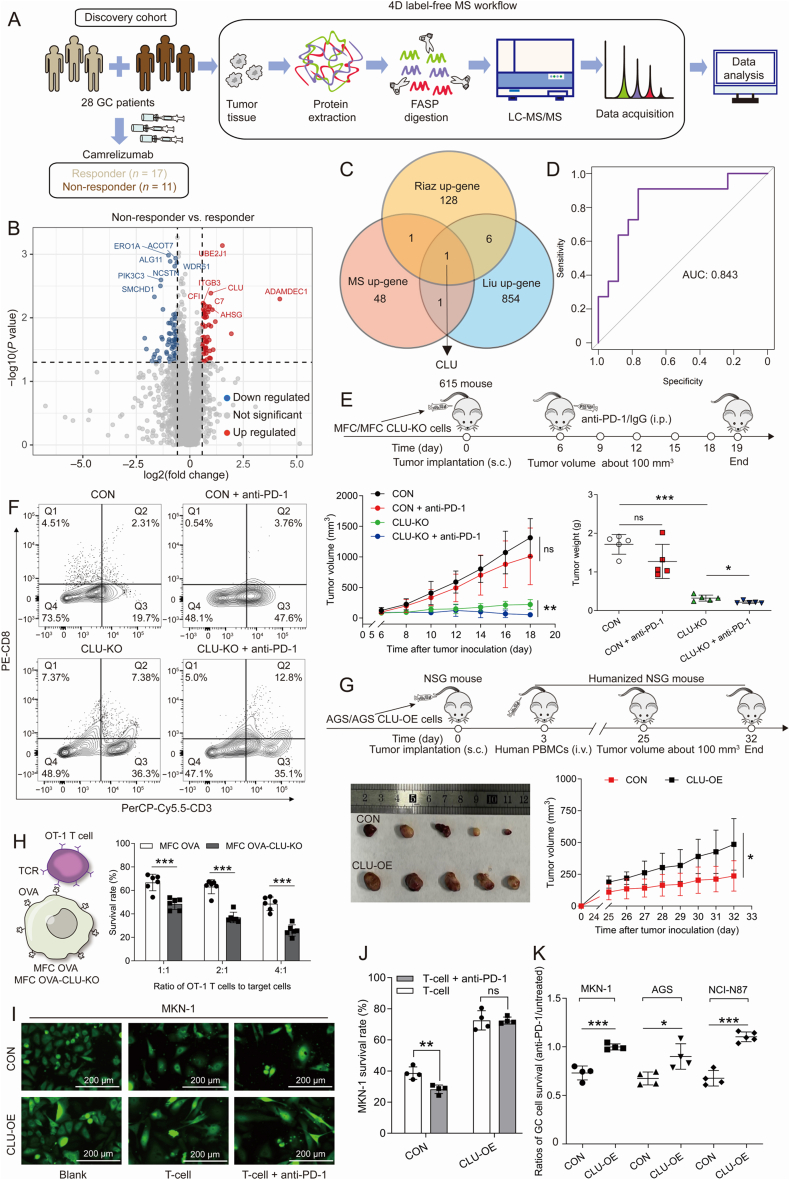
Clusterin (CLU) is a potential negative predictive biomarker for anti-programmed death-1 (PD-1) immunotherapy in gastric cancer (GC). (A) Proteomics workflow: tumor samples from GC patients were collected prior to surgery. Samples from 17 responders and 11 non-responders were analyzed using the four-dimensional (4D) label-free mass spectrometry (MS) method. (B) Volcano plot showing changes in protein abundance non-responders vs. responders. Colored dots represent proteins that pass the threshold of > 1.5-fold change and *P* < 0.05. (C) Venn analysis of three datasets of non-responders vs. responders up-regulated genes; dataset Nos.: GSE91061 (Riaz) and pht002589.v3.p1 (Liu). (D) Receiver operating characteristic (ROC) curve analysis of CLU in MS data for predicting the efficacy of anti-PD-1 immunotherapy in GC. (E) Comparison of tumor volume and weight in the control (CON) group, CON + anti-PD-1 group, CLU knock out (CLU-KO) group, and CLU-KO + anti-PD-1 group in 615 mice (*n* = 5). (F) Representative flow cytometric maps of CD8^+^ T cells in four groups of tumor samples (*n* = 5). (G) Schematic diagram of humanized NSG mouse model, and the tumor size and volume in the CON and CLU over expression (CLU-OE) groups (*n* = 5). (H) Pre-activated OT-1 T cells were co-cultured with mouse forestomach carcinoma (MFC) and MFC ovalbumin (OVA) -CLU-KO cells, and GC cells survival rate was assessed using CCK-8 assays (*n* = 6), 1:1, 2:1, and 4:1 ratio of OT-1 T cells to target cells in co-incubation. (I) Representative fluorescence images of different groups in MKN-1 co-culture system with T cells. (J) CLU-OE or CON MKN-1 cells were co-cultured with T cells treated with or without anti-PD-1, followed by quantification of GC cell survival. (K) Ratios of GC cell survival (anti-PD-1/untreated) across experimental groups in CLU-OE models. All values were presented as the mean ± SD. ^∗^*P* < 0.05, ^∗∗^*P* < 0.01, ^∗∗∗^*P* < 0.001; ns, not significant. FASP: filter aided sample prep; LC-MS: liquid chromatograph mass spectrometer; AUC: area under curve; PBMCs: peripheral blood mononuclear cells; s.c.: subcutaneous injection; i.p.: intraperitoneal injection; i.v.: intravenous injection; TCR: T cell receptor.

Functional studies further demonstrated the role of CLU in regulating the tumor immune response. In syngeneic GC mouse models, CLU knockout (CLU-KO) markedly inhibited tumor growth and significantly enhanced the efficacy of anti-PD-1 therapy (Figs. 1E and S3A–C). Compared with control tumors, CLU-KO tumors showed smaller volumes and weights (Fig. 1E), and reduced Ki-67 staining, indicating suppressed tumor proliferation (Fig. S3D). Kyoto Encyclopedia of Genes and Genomes (KEGG) analysis revealed enrichment of the complement and coagulation cascade in non-responders, pathways known to influence immune cell activity (Fig. S3E). Single sample gene set enrichment analysis further showed that responders had higher CD8^+^ T-cell infiltration, underscoring the importance of T cells in anti-PD-1 efficacy (Fig. S3F). Consistently, in T-cell-deficient NOD-SCID mice, the tumor-suppressive effect of CLU ablation was largely lost, with only minor growth reduction observed (Figs. S3G–I). In conclusion, the absence of functional T cells mitigated the effect of CLU abrogation on tumor growth inhibition, suggesting a critical role for T cells. Importantly, CLU abrogation markedly increased CD8^+^ and CD4^+^ T-cell infiltration in 615 mice tumor model. Notably, CLU loss further enhanced CD8^+^ T-cell infiltration when combined with PD-1 blockade, while CD4^+^ T cells showed a modest but consistent increase (Figs. 1F and S4). Splenic analysis confirmed elevated T-cell populations following CLU abrogation, indicating both local and systemic immune activation (Fig. S5). Besides, IHC staining confirmed enhanced intratumoral T-cell populations (Figs. S6A and B), and enzyme-linked immunosorbent assays demonstrated increased secretion of interferon-γ (IFN-γ) and interleukin-2 (IL-2) in the TME (Figs. S6C and D). On the other hand, CLU overexpression (CLU-OE, Fig. S6E) accelerated tumor growth in a humanized immunity GC model (Figs. 1G, S6F and S6G). These results clarify the observed differences across models. In T-cell–competent syngeneic mice, CLU-KO elicits a strong T-cell–mediated antitumor response, creating a ceiling effect that limits additional benefit from PD-1 blockade. In contrast, this effect is largely lost in NOD-SCID mice, where the absence of functional T cells abrogates the immune contribution. Overall, the diminished efficacy of CLU-KO in T-cell–deficient settings confirms that its antitumor effects are predominantly T-cell dependent.

*In vitro* experiments further supported these observations. Ovalbumin (OVA)-specific OT-1 T cells were co-cultured with OVA-expressing GC cells (Figs. S7A and B), and CLU ablation significantly enhanced T-cell cytotoxicity, indicating that high CLU expression enables tumor cells to evade T-cell killing (Fig. 1H). Across multiple GC cell lines, CLU emerged as a stronger negative biomarker for predicting anti-PD-1 efficacy than PD-L1. MKN-1, AGS, and NCI-N87 were selected for functional studies due to their inverse CLU/PD-L1 expression patterns (Figs. S7C and D), where assays confirmed that high CLU expression promoted tumor survival and reduced IFN-γ/tumor necrosis factor-alpha (TNF-α) secretion (Figs. S7E, S7F and S8), while low CLU expression restored T-cell cytotoxicity (Fig. S9). Minimal changes in inherently CLU-low NCI-N87 cells supported the specificity of CLU-dependent regulation (Fig. S9). To evaluate the predictive value of CLU across diverse GC contexts, five cell lines (BGC-823, MKN45, MKN-1, AGS, NCI-N87) with descending CLU expression were analyzed. Intriguingly, while lower CLU expression in GC cells generally correlated with reduced tumor survival in co-culture systems, a biphasic pattern (initial survival increase followed by decline) was observed (Fig. S10A). T-cell responsiveness, measured by the viability ratio (anti-PD-1/untreated), also mirrored this biphasic trend (Fig. S10B), suggesting that contextual factors may influence CLU's predictive utility. Functional assays further confirmed that CLU-OE in GC cells consistently suppressed T-cell cytotoxicity and reduced the efficacy of anti-PD-1 therapy (Figs. 1I–K and S11), while CLU silencing enhanced T-cell activity and improved treatment response (Fig. S12). Together, these results establish CLU as a critical regulator of T-cell function and a robust negative biomarker for predicting anti-PD-1 immunotherapy response in GC.

In summary, this study identifies CLU as a critical regulator of GC progression and immune evasion, exerting dual functions by promoting tumor proliferation and suppressing T-cell activity. Multi-omics analyses further revealed CLU as the only protein consistently upregulated in anti-PD-1 non-responders, outperforming PD-L1 as a predictive biomarker. These findings highlight the clinical potential of CLU inhibition to simultaneously restrain tumor growth and enhance immunotherapy efficacy. Nevertheless, mechanistic insights into CLU's immunomodulatory pathways, validation in larger patient cohorts, and integration with complementary biomarkers (e.g., TMB, and MSI) remain necessary. Overall, CLU represents a promising negative biomarker and therapeutic target that could improve patient stratification and guide more precise immunotherapy strategies in GC.

## CRediT authorship contribution statement

**Yu Zhang:** Writing – original draft, Methodology, Data curation, Formal analysis, Investigation. **Hao-Yi Zhu:** Methodology, Formal analysis, Visualization. **Cong-Cong Ma:** Methodology, Formal analysis, Visualization. **Ning Wang:** Resources, Project administration. **Yan Li:** Resources, Project administration. **Guo-Liang Lu:** Resources, Project administration. **Xing-Jie Dai:** Resources, Project administration. **Bo Wang:** Resources, Project administration. **MAA Mamun:** Formal analysis, Methodology, Visualization. **Ying Li:** Methodology, Conceptualization, Resources. **Xiao-Ying Zhao:** Methodology, Conceptualization, Resources. **Jing-Ru Pang:** Methodology, Conceptualization, Resources. **Ning-Jie Guo:** Methodology, Conceptualization, Resources. **Feng-Yu Qi:** Conceptualization, Methodology, Resources. **Jian-Gang Sun:** Resources, Project administration. **Hong-Min Liu:** Project administration, Resources, Funding acquisition. **Feng-Wu Liu:** Resources, Project administration. **Piet Herdewijn:** Writing – review & editing, Supervision, Conceptualization, Project administration, Resources. **Long-Fei Zhao:** Writing – review & editing, Supervision, Conceptualization, Project administration, Resources. **Yi-Chao Zheng:** Writing – review & editing, Supervision, Resources, Funding acquisition, Conceptualization, Project administration.

## Ethical statement

The study was approved by the Research Ethics Committee of the First Affiliated Hospital of Zhengzhou University (Approval No.: 2020-KY-386). Participants gave informed consent to participate in the study before taking part. The animal study was approved by the Zhengzhou University Animal Experimentation Ethics Committee (Approval No.: 24-IACVC-Y070). Human GC cell lines NCI-N87, MKN-45, MKN-1, MGC-803, HGC-27, BGC-823, AGS, and mouse GC cell line MFC were purchased from the National Cellular Resource Center (Beijing, China).

## Declaration of competing interest

The authors declare that they have no known competing financial interests or personal relationships that could have appeared to influence the work reported in this paper.
