# Supplementary material for: Multiple gastric cancer tissue proteomic identification predicts CLU as a biomarker for anti-PD-1 immunotherapy
Source: J Pharm Anal. 2026 Apr 1;16(7):101615. doi: 10.1016/j.jpha.2026.101615 (PMC13380152; doi:10.1016/j.jpha.2026.101615)
Supplement: Multimedia component 1 [file mmc1.docx]

***Supporting Information***

**Multiple gastric cancer tissue proteomic identification predicts CLU as a biomarker for anti-PD-1 immunotherapy**

**1. Material and methods**

**1.1 Clinical cohort**

Ethical approval for this study was granted by the First Affiliated Hospital of Zhengzhou University, Henan Province, China (2020-KY-386). We prospectively collected samples from 28 patients with advanced gastric cancer (GC) who underwent surgical resection. Only samples containing more than 60% tumor content were included, and all pathological assessments were independently reviewed by a senior pathologist. Tumor biopsy tissues from all GC patients were obtained prior to treatment with the camrelizumab [1]. Patients were divided into two groups based on their best overall response to camrelizumab, according to the response evaluation criteria in solid tumors: (a) responders, including complete response (CR) and partial response (PR), and (b) non-responders, including disease progression (PD) and stable disease (SD).

**1.2 Tumor tissue collection and proteomic analysis in GC patients**

Tumor tissues from surgically resected GC patients were collected and gently washed with pre-cooled phosphate buffer saline (PBS) to remove surface blood. The tissues were then fixed in 10% formalin and embedded in paraffin wax. A total of 28 GC tissue samples were collected. Proteomic experiments were conducted by Jingjie PTM BioLab Ltd. (Hangzhou, Zhejiang, China). After protein extraction, trypsin digestion, and quality control analysis, 4D label-free quantitative analysis was performed to identify significantly differentially expressed proteins. Finally, downstream bioinformatics analysis was conducted.

**1.3 Experimental Workflow: Protein Extraction, Digestion, and LC-MS/MS Analysis**

Tumor tissue samples from 28 GC patients were dewaxed, lysed with sodium dodecyl sulfate buffer containing protease inhibitors, and subjected to ultrasonic disruption. After centrifugation to remove tissue debris, equal amounts of protein from each sample were precipitated with acetone, washed, and resuspended in Triethylammonium bicarbonate (TEAB) buffer. The proteins were digested with trypsin overnight, followed by reduction with DTT and alkylation with IAA. The resulting peptides were separated using a NanoElute UHPLC system with a gradient elution (mobile phase A: 0.1% formic acid, 2% acetonitrile; mobile phase B: 0.1% formic acid, 100% acetonitrile). The peptides were ionized via a Capillary Ion Source and analyzed using a timsTOF Pro mass spectrometer in PASEF mode, with parent ions and fragments detected in high-resolution TOF. The MS settings included an ion source voltage of 1.6 kV, a secondary mass spectrum scanning range of 400–1500 m/z, and dynamic exclusion for tandem mass scans to avoid redundancy.

**1.4 Cell Culture**

Human GC cell lines NCI-N87, MKN-45, MKN-1, MGC-803, HGC-27, BGC-823, AGS, and mouse GC cell line MFC were purchased from the National Cellular Resource Center. All cell lines were authenticated by short tandem repeat (STR) analysis. Cells were cultured in RPMI-1640 medium (KeyGEN BioTECH, Nanjing, Jiangsu, China) supplemented with 10% fetal bovine serum (FBS, Royacel, Lanzhou, Gansu, China), except for the MGC-803 cell line, which was cultured in DMEM medium (KeyGEN BioTECH, Nanjing, Jiangsu, China). All cultures were maintained in a cell incubator at 37°C with 5% CO_2_.

**1.5 Antibodies and reagents**

PD-L1 (13684S) Rabbit mAb, CLU (42143S) Rabbit mAb, Ki-67 (9449T) Mouse mAb, CD4 (25229T) Rabbit mAb, CD8α (98941T) Rabbit mAb and CLU (23159S) Rabbit mAb were purchased from Cell Signaling Technology (Danvers, Massachusetts, USA). Chicken OVA (ab306591) Rabbit mAb was purchased from Abcam (Cambridge, UK). Human/mouse GAPDH (AB-P-R 001) was purchased from GOODHERE BIOTECH (Hangzhou, Zhejiang, China). PerCP/Cyanine5.5 anti-mouse CD3 (100217), APC anti-mouse CD4 (100516), PE anti-mouse CD8α (100708) and PE/Cyanine7 anti-mouse CD45 (103114) were purchased from Biolegend (San Diego, California, USA). Anti-mouse CD279 (P377) was purchased from Leinco Technologies, Inc (St. Louis, Missouri, USA), and Camrelizumab was purchased from Hengrui Pharmaceuticals Co., Ltd (Lianyungang, Jiangsu, China).

**1.6 Establishment of CLU-knock out (KO)/overexpression (OE) and MFC-Ovalbumins (OVA) cell lines**

MFC cells were transfected with lentiviral vector (LV)-CLU-sgRNA or empty LV (GeneChem, Shanghai, China), then screened with puromycin in continuous culture for 5 days. single cloned cell was selected by dilution to establish stable MFC CLU-KO or control (CON) cell lines. The CLU-KO sgRNA sequence were: GGAGATTCAGAACGCCGTCC, and the CON sequence were: CGCTTCCGCGGCCCGTTCAA. Similarly, MKN-1, AGS, and NCI-N87 cells were transfected with LV5-CLU-Homo or empty LV (GenePharma, Suzhou, Jiangsu, China) to generate stable CLU-OE or CON cell lines. For MFC-OVA cells, OVA oligonucleotides were cloned into pLVX-IRES-Zsgreen1 vector and packaged into lentiviruses according to published protocols[2]. The same protocol was used to construct the MFC-OVA.

**1.7 Peripheral blood cells (PBMCs) separation and activation**

Peripheral blood from 3-4 healthy human volunteers was used in this study. PBMCs were separated from human peripheral blood using Lymphocyte Separation Medium (Solarbio, Beijing, China), following the manufacturer’s protocols. The PBMCs were allowed to rest for 2 hours in a petri dish containing RPMI-1640 complete medium, with gentle shaking to collect the floating PBMCs from the supernatant. For activation, PBMCs were stimulated with ImmunoCult™ Human CD3/CD28 T Cell Activator (STEMCELL Technologies, Vancouver, British Columbia, Canada) for 72 hours. After activation, cell counts were collected for subsequent experiments.

**1.8 OT-1 cells separation and activation**

Single nucleated cells were separated from the spleens of OT-1 mice using the Mouse Spleen Single Nucleated Cell Isolation Solution Kit (Solarbio, Beijing, China), following the manufacturer’s protocols. CD8+ T cells were then separated using the EasySep™ Mouse CD8+ T Cell Isolation Kit (STEMCELL Technologies, Vancouver, British Columbia, Canada) to obtain CD8+ OT-1 cells. These cells were subsequently activated for 72 hours using the ImmunoCult™ Mouse T Cell Activator Kit (STEMCELL Technologies, Vancouver, British Columbia, Canada).

**1.9 Killing experiment**

For the killing experiment, 2,000 target tumor cells were inoculated into 96-well plates, and RPMI 1640 complete medium was added, supplemented with 10 ng/mL of IL-2 to co-culture with activated T cells. Effector: Target ratio: (human T cells: human tumor cells were 1:1, 5:1, and 10:1, OT-1 cells: MFC-OVA cells were 1:1, 2:1, and 4:1). After co-cultivation for 24, 48, and 72 hours, the culture supernatant was discarded, and the cells were rinsed twice with PBS. Tumor cell activity was then assessed using the CCK8 assay (YEASEN, Shanghai, China) according to the manufacturer’s instructions.

**1.10 Enzyme linked immunosorbent assay (ELISA)**

For human interferon-γ (IFN-γ) and tumor necrosis factor-α (TNF-α) assays, cytokines were detected in the supernatants of tumor cells co-incubated with T cells using an ELISA kit (MULTI SCIENCES, Hangzhou, Zhejiang, China) according to the manufacturer’s instructions. For mouse IFN-γ and TNF-α assays, equal weights of mouse tumor tissues were homogenized in PBS using a tissue homogenizer, and cytokine concentrations in the supernatants were measured using an ELISA (Thermo Fisher Scientific, Waltham, Massachusetts, USA). Concentrations were calculated against a standard curve and expressed as ng/mL.

**1.11 Western blotting**

Cells were collected and lysed in RIPA cell lysis buffer containing protease inhibitor cocktail (MeilunBio, Dalian, Liaoning, China). Proteins were separated on 8-12% SDS-polyacrylamide gels. Immunoblot was performed according to the standard protocol described previously[3].

**1.12 Immunohistochemical staining (IHC)**

Patient tumor tissues and mouse tumor fragments were fixed in 10% formalin solution for 48h and embedded in paraffin. The paraffin blocks were sectioned to a thickness of 5 µm. Sections were deparaffinized in xylene and then dehydrated through a series of graded ethanol. Antigen retrieval was performed using ethylenediaminetetraacetic acid (EDTA) or citrate, followed by blocking of endogenous peroxidase with 3.5% H_2_O_2_. The sections were then blocked with 5% goat serum. Overnight incubation at 4°C was carried out with primary antibodies against CLU, PD-L1, CD4, CD8, and Ki-67. Afterward, sections were incubated with secondary antibodies for 2 hours at room temperature and stained using a DAB kit (Beyotime, Shanghai, China). Following staining, sections were digitally scanned with an Aperio AT2 scanner (Leica Biosystems, Wetzlar, Germany) and analyzed using the Aperio Image Analysis Workstation (Leica Biosystems, Wetzlar, Germany) with pathologist-trained nuclear, membrane, and cytoplasmic algorithms. Protein expression was assessed based on H-Scores obtained from the Aperio Image Analysis Workstation. Detailed information on the antibodies used is provided in the Antibodies and Reagents section.

**1.13 Mouse lymphocyte isolation and flow cytometry**

Mouse lymphocyte surface markers were analyzed using the following antibodies: PerCP/Cyanine5.5-CD3, APC-CD4, PE-CD8α, and PE/Cyanine7-CD45. Detailed information on these antibodies is provided in the Antibodies and Reagents section.

Single-cell suspensions were prepared as described in standard protocols[4]. Mice were euthanized, and tumor tissues were excised. Tissues were digested in a solution containing 1 mg/mL collagenase IV (Solarbio, Beijing, China), 0.2 mg/mL hyaluronidase (Sigma, St. Louis, Missouri, USA), and DNase I (Sigma, St. Louis, Missouri, USA) at 37°C for 1 h, with gentle agitation every 15 min to ensure thorough digestion. The digestion was halted with FBS, and the mixture was filtered through a 70 μm filter. After centrifugation at 400 g for 5 min, cells were resuspended in staining buffer (2% FBS in PBS).

For spleen samples, spleens were transferred to a petri dish, PBS buffer was added, and spleens were gently ground. The cell suspension was then filtered through a 70 μm filter, transferred to a centrifuge tube, and treated with erythrocyte lysis solution (Elabscience, Wuhan, Hubei, China) for 15 min. PBS was added to stop the lysis reaction, and cells were centrifuged at 400 g for 5 min before resuspending in staining buffer.

For cell staining, cells were grouped and stained according to the manufacturer’s instructions, with antibodies added for 30 min at 4°C in the dark. Cells were finally resuspended in staining buffer and analyzed by flow cytometry (BD Biosciences, Franklin Lake, New Jersey, USA). Data analysis was performed using FlowJo software.

**1.14 Cell fluorescence imaging**

Fluorescence imaging was conducted using a fluorescence microscope (Nikon, Tokyo, Japan). Briefly, each experimental group was imaged at the end of the experiment, utilizing the EGFP tags incorporated during the construction of the CLU-OE cell line for visualization.

**1.15 Transfection of siRNAs**

Target cells were seeded in medium dishes (4×10^5^ cells per dish). After 12 hours, they were transfected with siRNA targeting CLU (GenePharma, Suzhou, Jiangsu, China) using Entranster^TM^-H4000 (Engreen Biosystem, Beijing, China) following the manufacturer's instructions. Cells were collected 48 hours post-transfection for western blot analysis and additional experiments. The sequences for both CLU-targeting siRNA and the negative control siRNA (siNC) were as follows: si-NC: 5’-UUCUCCGAACGUGUCACGUTT-3’; si-CLU: 5’-CCAGACGGUCUCAGACAAUTT-3’.

**1.16 In vivo tumor model and drug treatment regimen**

Animal experiments were approved by the Zhengzhou University Animal Experimentation Ethics Committee (Approval number: 24-IACVC-Y070), and the experimental procedures were conducted in strict compliance with the regulations of the Laboratory Animal Management Code. 6-8-week-old 615 and NOD-SCID mice were purchased from the Institute of Hematology, Chinese Academy of Medical Sciences, and from Hunan SJA Laboratory Animal Center, Inc. 3-4-week-old NSG mice were purchased from Shanghai Model Organisms Center, Inc. Prior to the experiments, all purchased mice were housed in the Specific Pathogen Free (SPF) Animal Facility at Zhengzhou University for one week to acclimatize to the housing conditions.

In the 615 mice model, 1×10^6^ MFC or MFC CLU-KO cells were injected subcutaneously. When tumors reached approximately 100 mm³ in volume, mice were randomly assigned into four groups based on tumor size: control (CON), control with anti-PD-1 (CON+anti-PD-1), CLU-KO, and CLU-KO with anti-PD-1 (CLU-KO+anti-PD-1). Anti-mouse PD-1 antibody (200 μg, Leinco Technologies, Inc., USA) or control PBS was administered intraperitoneally every three days, for a total of five doses. Tumor volume and body weight were measured every two days, with tumor volume calculated as (length × width²) × 0.5.

In the NOD-SCID mice model, procedures followed those described in the 615 mice model construction. Mice were divided into two groups: CON and CLU-KO. Each mouse was inoculated with 1×10^6^ cells, and tumor volume was measured every two days for a total of five measurements.

In the humanized NSG mice model, humanized mice were generated as previously described in the literature[5-7]. Each mouse was subcutaneously injected with 1×10^7^ AGS or AGS CLU-OE cells. Three days post-inoculation, mice were randomly divided into two groups based on tumor volume. PBMCs from healthy volunteers were adjusted to a density of 5×10^6^ cells/100 μL, and each mouse received a slow tail vein injection of 200 μL of the PBMC suspension. Starting one week after PBMCs transplantation, 100 μL of peripheral blood was collected weekly for humanization analysis using flow cytometry (Human-CD45). Tumor volume was measured daily for a total of eight measurements.

**1.17 Timer 2.0 database analysis**

Timer 2.0 (http://timer.cistrome.org/) was used to analyze tumor-infiltrating cells in the tumor microenvironment (TME), following the published protocol[8, 9]. CLU and the corresponding immune cell types were entered into the 'Relationship between Immune Infiltration and Gene Expression' module. The output was generated using six algorithms: TIMER, XCell, MCP-counter, CIBERSORT, EPIC, and quanTIseq.

**1.18 RNA-sequence (RNA-seq) Data Processing and Differential Expression Analysis**

The raw gene count data used in this study were obtained from the Gene Expression Omnibus (GEO) database and two previously published studies involving metastatic melanoma patients treated with anti–PD-1 immunotherapy[10, 11]. Date was saved in GEO database (GSE91061) and <https://www.ncbi.nlm.nih.gov/projects/gap/cgi-bin/study.cgi?study_id=phs000452.v3.p1>. To identify differentially expressed genes (DEGs) between groups, we employed the DESeq2 package, a widely used R package designed for differential analysis of count data from RNA-seq experiments[12]. To control the false discovery rate (FDR), genes with an adjusted p-value < 0.2 were considered to differentially expressed.

**1.19 Immune cell abundance inference**

We selected immune-related signatures of immune cell types from Charoentong's study[13]. Gene set variation analysis (GSVA) package[14] with single sample gene set enrichment analysis method[15] was used to infer the relative abundance of immune cell infiltration in the tumor using protein intensity. We employed unpaired one-sided Student's t-test to examine differences between responders and non-responders.

**1.20 Data & Code Availability**

All R code used for the analyses in this study is publicly available in my GitHub repository: <https://github.com/longfei8533/CLU_biomarker_anti-PD1>.

**1.21 Statistical analysis**

The relationships between treatment response and clinical-pathological features were evaluated using the Wilcoxon test for continuous variables, and Fisher's exact test for categorical variables. We use the two-sided Student's t-test to evaluate the difference of log2-transformed protein intensity between groups. For two-group comparisons, two-sided Student's t-tests were performed in GraphPad Prism v6.0, while multiple-group comparisons were conducted using one-way analysis of variance (ANOVA). The data are presented as mean ± SD from at least three independent experiments. Statistical significance was determined using two-sided p-values, with differences considered significant at ^*^*P* < 0.05, ^**^*P* < 0.01, and ^***^*P* < 0.001, unless otherwise indicated. The R “clusterProfiler” package was used for kyoto encyclopedia of genes and genomes (KEGG) pathway enrichment analyses[16].

**2. Supplemental Figures**

**Fig. S1.** Proteomic and RNA-seq analysis of differentially expressed genes in the non-responder and responder groups. (A, B) Volcano plots showing changes in protein abundance between non-responders and responders in RNA-seq from Liu (A) and Riaz (B). Red, up-regulated; Blue, down-regulated; Gray, non-significant. (C) Proteomic and RNA-seq p-values and log_2_Fold changes for CLU in non-responders *vs* responders. (D) Venn analysis of down-regulated genes in non-responders *vs* responders based on proteomic and RNA-seq.

**Fig. S2.** CLU is a stratifying biomarker for anti-PD-1 immunotherapy in GC patients and is closely related to tumor immune-infiltrating cells. (A, B) Representative images of IHC (A) and H-score for CLU and PD-L1 (B) in tumor tissues from 28 GC patients. (C) The relationship between tumor-infiltrating immune cells and CLU expression in the TME of 415 GC patients. All values were presented as the mean ± SD. ^**^, *P* <0.01; ns, not significant. N: negative; +: degree of positivity; CLU: clusterin; PD-L1: programmed cell death ligand 1; CAFs: cancer-associated fibroblasts; NK cells: natural killer cells; Tregs: regulatory T cells.

**Fig. S3.** CLU abrogation enhanced the efficacy of anti-PD-1 therapy in the GC mouse model by inhibiting tumor proliferation and enhancing T cell function. (A) CLU expression in control and CLU-KO MFC cells. (B) Comparison of tumor size in the CON group, CON + anti-PD-1 group, CLU-KO group, and CLU-KO + anti-PD-1 group in 615 mice (*n* = 5). (C) Body weight of 615 mice in CON group, CON + anti-PD-1 group, CLU-KO group, CLU-KO + anti-PD-1 group (*n* = 5). (D) Representative images of IHC analysis for Ki-67 expression in tumor tissues of the four group, with H-scores (*n* = 5). (E) KEGG pathway enrichment analysis of differentially expressed proteins in proteomics (non-responders *vs* responders). (F) Immune cell infiltration in responders and non-responders. (G–I) Tumor size (G), volume (H), and weight (I) in NOD-SCID mice of the CON and CLU-KO groups (*n* = 6). All values were presented as the mean ± SD. ^*^, *P* <0.05; ^***^, *P* <0.001; ns, not significant. N: negative; +: degree of positivity; CON: control; KO: knock out.

**Fig. S4.** CLU abrogation increased the infiltration ratio of CD8 and CD4^+^ T cells in the tumors of 615 mice. (A, B) Representative flow cytometric maps of CD4+ T cells and the statistics analysis of CD8^+^ (A) and CD4^+^ T cells (B) in four groups of tumor samples (*n* = 5). All values were presented as the mean ± SD. ^*^, *P* <0.05; ^***^, *P* <0.001; ns, not significant. CON: control; KO: knock out.

**Fig. S5.** CLU abrogation stimulates the immune response of the spleen. (A–D) Representative flow cytometry results of CD8^+^ (A) and CD4^+^ T cells (B) and the statistics analysis of CD8^+^ (C) and CD4^+^ T cells (D) in four groups from spleen samples (*n* = 5). All values were presented as the mean ± SD. ^*^, *P* <0.05; ^**^, *P* <0.01; ns, not significant. CON: control; KO: knock out.

**Fig. S6.** CLU abrogation increased the infiltration ratio of CD8 and CD4+ T cells in the tumors of 615 mice, as well as the levels of IFN-γ and IL-2. (A, B) IHC of CD8 (A) and CD4 (B) expression in tumor tissues from the four groups, with H-scores (*n* = 5). (C, D) ELISA analysis of IFN-γ (C) and IL-2 (D) expressions in tumors from different groups (*n* = 5). (E) CLU expression in control and CLU-OE AGS cells. (F) Populations of human CD45+ cells in the peripheral blood from the humanized mouse following different treatments. (G) Tumor weight in the CON and CLU-OE groups (*n* = 5). All values were presented as the mean ± SD. ^*^, *P* <0.05; ^**^, *P* <0.01; ^***^, *P* <0.001; ns, not significant. CON: control; KO: knock out; OE: over expression; PBMC; peripheral blood mononuclear cell.

**Fig. S7.** CLU expression in GC cells impairs T-cell mediated tumor cell killing and reduces IFN-γ and TNF-α secretion. (A) OT-1 mice genotype validation: OT-1: 350 bp, Positive control: 521 bp. (B) OVA expression in overexpressed and control MFC cells, and CLU expression levels in CLU KO and control MFC cells. (C, D) CLU and PD-L1 expressions in seven GC cell lines (*n* ≥ 3). (E) MKN-1, AGS, and NCI-N87 cells were co-cultured with pre-activated T cells for 72 hours. Surviving GC cells were analyzed using CCK-8 assays (*n* = 4). (F) ELISA analysis of IFN-γ and TNF-α secretion at 10:1 ratio in different co-incubation systems (*n* = 3). All values were presented as the mean ± SD. ^*^, *P* <0.05; ^**^, *P* <0.01; ^***^, *P* <0.001; ns, not significant.

**Fig. S8.** CLU-OE in GC cells can weaken the cytotoxic effect of T cells against tumor cells, and reduce the secretion of IFN-γ and TNF-α. (A, B) CLU-OE in three GC cell lines (A) and survival rate (B) of MKN-1, AGS, and NCI-N87 cells at different E:T ratios after 72 hours of co-incubation (*n* = 4). (C) ELISA analysis of IFN-γ and TNF-α secretion in different co-incubation systems at a 10:1 E:T ratio (*n* = 3). All values were presented as the mean ± SD. ^*^, *P* <0.05; ^**^, *P* <0.01; ^***^, *P* <0.001; ns, not significant. CON: control; OE: overexpression.

**Fig. S9.** CLU-KD in GC cells can enhance the cytotoxic effect of T cells against tumor cells, and promote the secretion of IFN-γ and TNF-α. (A, B) CLU-KD in three GC cell lines (A) and survival rate (B) of MKN-1, AGS, and NCI-N87 cells at different E:T ratios after 72 hours of co-incubation (*n* = 4). (C) ELISA analysis of IFN-γ and TNF-α secretion in different co-incubation systems at a 10:1 E:T ratio (*n* = 3). All values were presented as the mean ± SD. ^*^, *P* <0.05; ^**^, *P* <0.01; ^***^, *P* <0.001; ns, not significant. CON: control; KD: knock down.

**Supplementary Fig. S10.** CLU expression in GC cells reduces therapeutic efficacy by attenuating the T cell response to anti-PD-1 immunotherapy. (A) Preactivated T cells were co-cultured with various GC cells, treated with PD-1 antibody, and GC cell survival was assessed. (B) The ratio of cell survival after PD-1 antibody treatment compared to before treatment in the different groups (*n* ≥ 3). All values were presented as the mean ± SD. ^*^, *P* <0.05; ^**^, *P* <0.01; ns, not significant.

**Fig. S11.** CLU-OE in GC cells reduces therapeutic efficacy by attenuating the T cell response to anti-PD-1 immunotherapy. (A, B) Representative fluorescence images of different groups in AGS (A) and NCI-N87 (B) co-culture system with T cells, and the CLU- OE or control AGS and NCI-N87 cells were co-cultured with T cells treated with or without anti-PD-1, followed by quantification of GC cell survival (*n* ≥ 3). All values were presented as the mean ± SD. ^*^, *P* <0.05; ^**^, *P* <0.01; ns, not significant. CON: control; OE: overexpression.

**Fig. S12.** CLU-KD in GC cells enhance therapeutic efficacy by promote the T cell response to anti-PD-1 immunotherapy. (A) CLU-KD or control MKN-1, AGS, and NCI-N87 cells were co-cultured with anti-PD-1-treated T cells, followed by quantification of GC cell survival. (B) Ratios of GC cell survival (anti-PD-1/untreated) across experimental groups in CLU-KD models (*n* ≥ 3). All values were presented as the mean ± SD. ^**^, *P* <0.01; ^***^, *P* <0.001; ns, not significant. CON: control, KD: knock down.

**3. Supplemental table.**

| Table S1. Baseline demographics and clinical characteristics of 28 GC patients. | | | | |
| --- | --- | --- | --- | --- |
|  | Level | Responder (CR, PR) | Non-Responder (PD, SD) | *P*-value |
| Number | / | 17 | 11 |  |
| Gender (%) | Female | 6 (35.3) | 4 (36.4) | 1 |
|  | Male | 11 (64.7) | 7 (63.6) |  |
| Age (median) | / | 59 | 62 | 0.66 |
| T (%) | 1 | 1 (5.9) | 0 (0) | 0.36 |
|  | 2 | 3 (17.6) | 0 (0) |  |
|  | 4 | 13 (76.5) | 11 (100) |  |
| N (%) | 0 | 7 (41.2) | 1 (9.1) | 0.22 |
|  | 1 | 5 (29.4) | 3 (27.3) |  |
|  | 2 | 1 (5.9) | 3 (27.3) |  |
|  | 3 | 4 (23.5) | 4 (36.4) |  |
| M (%) | 0 | 5 (29.4) | 1 (9.1) | 0.36 |
|  | 1 | 12 (70.6) | 10 (90.9) |  |
| Degree of differentiation (%) | Moderate-poorly differentiated | 7 (41.2) | 4 (36.4) | 0.61 |
|  | Moderate differentiated | 4 (23.5) | 1 (9.1) |  |
|  | Poorly differentiated | 6 (35.3) | 6 (54.5) |  |
| Lauren's criteria (%) | Diffuse | 7 (41.2) | 8 (72.7) | 0.2 |
|  | Intestinal | 6 (35.3) | 1 (9.1) |  |
|  | Mix | 4 (23.5) | 2 (54.5) |  |

GC: gastric cancer; CR: complete response; PR: partial response; PD: disease progression; SD: stable disease; T: tumor; N: node; M: metastasis.

**4. Discussion**

Although our study provides robust clinical and experimental evidence that CLU functions as a negative predictor of anti-PD-1 efficacy in GC, several limitations and future directions merit discussion. While genetic modulation (KO/OE) clearly demonstrated that CLU influences T-cell infiltration and antitumor activity, the precise molecular mechanisms remain incompletely defined. Previous studies have shown that CLU participates in extracellular chaperone networks and cellular stress regulation[17-19]. Building on this foundation, our findings further suggest that CLU may also be closely associated with components of the complement and coagulation pathways, which could plausibly influence antigen presentation, cytokine gradients, or effector T-cell recruitment. Future mechanistic work should therefore investigate (i) whether CLU affects tumor antigen processing and co-stimulatory signaling, (ii) whether CLU alters cytokine or chemokine networks that regulate T-cell trafficking, and (iii) potential crosstalk between CLU and complement/coagulation cascades suggested by our pathway analyses.

The relatively small clinical proteomic cohort limits generalizability. Although CLU was consistently identified across integrated datasets and validated experimentally, larger multi-center cohorts are required to confirm its predictive utility. Prospective validation should evaluate CLU across GC molecular subtypes and determine whether combining CLU with established biomarkers, such as PD-L1 expression, TMB, or MSI which can improve patient stratification and predictive accuracy[20, 21]. Modeling approaches in larger datasets will be necessary to quantify the incremental benefit of CLU over current biomarkers.

Although genetic perturbation studies support CLU as a functional regulator of immune responsiveness, pharmacological inhibition of CLU has not yet been explored due to the current absence of validated small-molecule CLU inhibitors. Existing CLU-targeting strategies, such as antisense oligonucleotides (OGX-011), have shown therapeutic promise in other disease settings but remain limited in bioavailability, stability, and clinical readiness[22-24]. Given the secreted and multifunctional nature of CLU, the development of potent and selective small-molecule inhibitors that can effectively disrupt CLU-mediated signaling represents a critical next step. Future work should focus on identifying druggable domains within CLU, screening for inhibitory compounds, and evaluating their use: alone or in combination with PD-1 blockade to determine whether pharmacological targeting of CLU can enhance antitumor immunity and support its translation from biomarker to therapeutic target.

Finally, the heterogeneity observed among CLU-high tumors suggests that CLU expression alone may not fully predict response. Integrating CLU with additional molecular or immune features may help uncover patient subgroups with distinct immunotherapy sensitivities. Despite these limitations, our multi-omics discovery, coupled with in vivo and in vitro functional validation, establishes CLU as a mechanistically plausible and clinically meaningful negative biomarker for anti-PD-1 therapy in GC. This work provides a foundation for future mechanistic dissection and large-scale clinical validation aimed at improving precision immunotherapy in this disease.

**5. Reference**

[1] A. Markham, S.J. Keam, Camrelizumab: First Global Approval, Drugs 79 (2019) 1355-1361 <https://doi.org/10.1007/s40265-019-01167-0>.

[2] G. Tiscornia, O. Singer, I.M. Verma, Production and purification of lentiviral vectors, Nat Protoc 1 (2006) 241-245.

[3] D.-D. Shen, J.-R. Pang, Y.-P. Bi, et al., LSD1 deletion decreases exosomal PD-L1 and restores T-cell response in gastric cancer, Mol. Cancer 21 (2022) 75 <https://doi.org/10.1186/s12943-022-01557-1>.

[4] X. Wang, L.-P. Hu, W.-T. Qin, et al., Identification of a subset of immunosuppressive P2RX1-negative neutrophils in pancreatic cancer liver metastasis, Nature Communications 12 (2021) 174 <https://doi.org/10.1038/s41467-020-20447-y>.

[5] S. Guedan, X. Chen, A. Madar, et al., ICOS-based chimeric antigen receptors program bipolar TH17/TH1 cells, Blood 124 (2014) 1070-1080 <https://doi.org/10.1182/blood-2013-10-535245>.

[6] P. De La Rochere, S. Guil-Luna, D. Decaudin, et al., Humanized Mice for the Study of Immuno-Oncology, Trends In Immunology 39 (2018) 748-763 <https://doi.org/10.1016/j.it.2018.07.001>.

[7] T. Ashizawa, A. Iizuka, C. Nonomura, et al., Antitumor Effect of Programmed Death-1 (PD-1) Blockade in Humanized the NOG-MHC Double Knockout Mouse, Clinical Cancer Research : an Official Journal of the American Association For Cancer Research 23 (2017) 149-158 <https://doi.org/10.1158/1078-0432.CCR-16-0122>.

[8] T. Li, J. Fu, Z. Zeng, et al., TIMER2.0 for analysis of tumor-infiltrating immune cells, Nucleic Acids Res 48 (2020) W509-W514 <https://doi.org/10.1093/nar/gkaa407>.

[9] T. Li, J. Fan, B. Wang, et al., TIMER: A Web Server for Comprehensive Analysis of Tumor-Infiltrating Immune Cells, Cancer Research 77 (2017) e108-e110 <https://doi.org/10.1158/0008-5472.CAN-17-0307>.

[10] N. Riaz, J.J. Havel, V. Makarov, et al., Tumor and Microenvironment Evolution during Immunotherapy with Nivolumab, Cell 171 (2017) <https://doi.org/10.1016/j.cell.2017.09.028>.

[11] D. Liu, B. Schilling, D. Liu, et al., Integrative molecular and clinical modeling of clinical outcomes to PD1 blockade in patients with metastatic melanoma, Nat Med 25 (2019) 1916-1927 <https://doi.org/10.1038/s41591-019-0654-5>.

[12] M.I. Love, W. Huber, S. Anders, Moderated estimation of fold change and dispersion for RNA-seq data with DESeq2, Genome Biol 15 (2014) 550.

[13] P. Charoentong, F. Finotello, M. Angelova, et al., Pan-cancer Immunogenomic Analyses Reveal Genotype-Immunophenotype Relationships and Predictors of Response to Checkpoint Blockade, Cell Rep 18 (2017) 248-262 <https://doi.org/10.1016/j.celrep.2016.12.019>.

[14] S. Hänzelmann, R. Castelo, J. Guinney, GSVA: gene set variation analysis for microarray and RNA-seq data, BMC Bioinformatics 14 (2013) 7 <https://doi.org/10.1186/1471-2105-14-7>.

[15] D.A. Barbie, P. Tamayo, J.S. Boehm, et al., Systematic RNA interference reveals that oncogenic KRAS-driven cancers require TBK1, Nature 462 (2009) 108-112 <https://doi.org/10.1038/nature08460>.

[16] T. Wu, E. Hu, S. Xu, et al., clusterProfiler 4.0: A universal enrichment tool for interpreting omics data, Innovation (Camb) 2 (2021) 100141 <https://doi.org/10.1016/j.xinn.2021.100141>.

[17] A. López Malizia, A. Merlotti, P.-E. Bonte, et al., Clusterin protects mature dendritic cells from reactive oxygen species mediated cell death, Oncoimmunology 13 (2023) 2294564 <https://doi.org/10.1080/2162402X.2023.2294564>.

[18] F. Zhang, M. Kumano, E. Beraldi, et al., Clusterin facilitates stress-induced lipidation of LC3 and autophagosome biogenesis to enhance cancer cell survival, Nature Communications 5 (2014) 5775 <https://doi.org/10.1038/ncomms6775>.

[19] X. Hu, Y. Ji, M. Zhang, et al., Targeting CXCL8 signaling sensitizes HNSCC to anlotinib by reducing tumor-associated macrophage-derived CLU, J Exp Clin Cancer Res 44 (2025) 39 <https://doi.org/10.1186/s13046-025-03298-7>.

[20] Y. Chen, K. Jia, Y. Sun, et al., Predicting response to immunotherapy in gastric cancer via multi-dimensional analyses of the tumour immune microenvironment, Nature Communications 13 (2022) 4851 <https://doi.org/10.1038/s41467-022-32570-z>.

[21] F. Sun, X. Gao, W. Wang, et al., Predictive biomarkers in the era of immunotherapy for gastric cancer: current achievements and future perspectives, Frontiers In Immunology 16 (2025) 1599908 <https://doi.org/10.3389/fimmu.2025.1599908>.

[22] F. Lamoureux, C. Thomas, M.-J. Yin, et al., Clusterin inhibition using OGX-011 synergistically enhances Hsp90 inhibitor activity by suppressing the heat shock response in castrate-resistant prostate cancer, Cancer Research 71 (2011) 5838-5849 <https://doi.org/10.1158/0008-5472.CAN-11-0994>.

[23] K.N. Chi, S.J. Hotte, E.Y. Yu, et al., Randomized phase II study of docetaxel and prednisone with or without OGX-011 in patients with metastatic castration-resistant prostate cancer, J Clin Oncol 28 (2010) 4247-4254 <https://doi.org/10.1200/JCO.2009.26.8771>.

[24] K.N. Chi, L.L. Siu, H. Hirte, et al., A phase I study of OGX-011, a 2'-methoxyethyl phosphorothioate antisense to clusterin, in combination with docetaxel in patients with advanced cancer, Clinical Cancer Research : an Official Journal of the American Association For Cancer Research 14 (2008) 833-839 <https://doi.org/10.1158/1078-0432.CCR-07-1310>.
